# Effects of a Synthetic Isoquinoline Derivative Against *Ichthyophthirius multifiliis* In Vivo and In Vitro in Grass Carp (*Ctenopharyngodon idella*)

**DOI:** 10.3390/pathogens14101069

**Published:** 2025-10-21

**Authors:** Xianqi Peng, Xialian Bu, Weisong Ma, Jinbiao Jiao, Xiaohong Huang, Yu Zhao, Jian Zhu, Lei Huang, Jing Chen, A’qin Zheng, Huantao Qu, Jiayun Yao

**Affiliations:** 1 Hubei Key Laboratory of Three Gorges Project for Conservation of Fishes, Chinese Sturgeon Research Institute, China Three Gorges Corporation, Yichang 443100, China; 2 Key Laboratory of Healthy Freshwater Aquaculture, Ministry of Agriculture and Rural Affairs, Key Laboratory of Fish Health and Nutrition of Zhejiang Province, Key Laboratory of Fishery Environment and Aquatic Product Quality and Safety of Huzhou City, Zhejiang Institute of Freshwater Fisheries, Huzhou 313001, China

**Keywords:** isoquinolines, *Ichthyophthirius multifiliis*, grass carp

## Abstract

White spot disease, caused by the parasitic *Ichthyophthirius multifiliis*, induces mortality exceeding 80% in intensive aquaculture systems, resulting in global annual losses of >$1 billion. Current chemical controls (e.g., formaldehyde) face environmental persistence and drug resistance challenges. This study developed a synthetic isoquinoline derivative, BHTCA (s)-2-tert-butoxycarbonyl-7-hydroxy-1,2,3,4-tetrahydroisoquinoline-3-carboxylic acid), as a biodegradable alternative against *I. multifiliis* in grass carp (*Ctenopharyngodon idella*). In vitro assays revealed stage-selective efficacy: theronts exhibited higher susceptibility (EC_50_ = 0.10 mg/L at 4 h) than tomonts (EC_50_ = 0.40 mg/L at 24 h), with BHTCA disrupting ciliary structures and compromising cyst integrity. In vivo trials (0.6 mg/L) reduced parasite loads by 78.1% and host mortality by 66.7% versus untreated controls. Acute toxicity tests confirmed a high therapeutic index (LC_50_ = 16.75 mg/L; >167× effective concentration). With demonstrated efficacy, low production cost, and minimal eco-toxicity, BHTCA presents a sustainable strategy for Ichthyophthiriasis management in aquaculture.

## 1. Introduction

Fish serve as a crucial protein source that is readily accessible and reliably abundant, particularly in developing countries [1]. Their availability plays a vital role in securing adequate nutrition and promoting human health [2].

*Ichthyophthirius multifiliis* poses a significant parasitic threat to nearly all species of freshwater fish due to its low host specificity [3]. These include channel catfish (*Ictalurus punctatus*) [4], rainbow trout (*Oncorhynchus mykiss*) [5], carp (*Cyprinus carpio* L.) [6], grass carp (*Ctenopharyngodon idella*) [7,8], and zebrafish (*Danio rerio*) [9]. *I. multifiliis* has been reported to affect 42% of channel catfish producers in the U.S., with over 2000 lb losses per producer [10], and to result in approximately USD 140 million annual losses for European trout farmers [11]. In China, this disease results in the an average mortality of 200,000 to 300,000 tons of fish annually, leading to economic losses averaging 2 to 3 billion RMB per year and inflicting severe damage on the Chinese aquaculture sector [12].

The life cycle of *Ichthyophthirius multifiliis* involves multiple stages, each characterized by unique morphological features and specific functional roles. The trophont, a feeding stage, inhabits the basal epidermal layers of the skin and gills of fish. After a period of growth, it detaches from the host and transforms into a tomont. This encysted stage undergoes repeated divisions, ultimately producing a large number of motile, infectious theronts. Upon contact with a fish host, the theront invades the epidermal tissue, where it develops into a trophont, thereby closing the cycle [13].

Mature parasites cause hyperplasia of epithelial tissue, macroscopically visible as 0.5–1.0 mm white spots [14]. Severe infections by *I. multifiliis* result in numerous white spots that significantly affect the respiration and osmoregulation of fish hosts, leading to massive mortality [15]. The reproductive process can be interrupted by eliminating either the free-swimming theront or the detached trophont through the application of antiprotozoal agents, thus limiting transmission of the parasite [13]. In the past, mercurous nitrate and malachite green, known for their carcinogenic and toxic properties to humans [15], were the primary treatments for ichthyophthiriosis in fish. However, these substances have since been banned due to their harmful effects. Instead, chemicals such as formaldehyde and trichlorfon were employed for short periods due to their initial effectiveness [16]; however, their application was discontinued owing to adverse impacts on the environment and potential risks to human health. Currently, no specific drugs are available to treat ichthyophthiriosis [12]. Consequently, there is an urgent need to develop environmentally friendly and safe therapeutic agents.

In order to ensure the sustainable growth of the aquaculture industry while maintaining food safety standards, research into the prevention and control of ichthyophthiriosis is of paramount importance. In light of these concerns, the development of plant-derived drugs for preventing and controlling ichthyophthiriosis holds considerable promise. Isoquinoline alkaloids, as a class of naturally derived compounds with broad biological activities, are found in families such as Papaveraceae and Berberidaceae. Such research not only contributes to the health and sustainability of the aquaculture industry but also addresses critical issues related to food safety and environmental impact. Consequently, this study aimed to evaluate the efficacy and safety of a novel synthetic isoquinoline derivative, (s)-2-tert-butoxycarbonyl-7-hydroxy-1,2,3,4-tetrahydroisoquinoline-3-carboxylic acid (BHTCA), against *I. multifiliis* in both in vitro and in vivo settings using grass carp as a model, to assess its potential as a sustainable treatment for ichthyophthiriasis.

## 2. Materials and Methods

### 2.1. Drug Composition and Preparation

**Figure 1:**
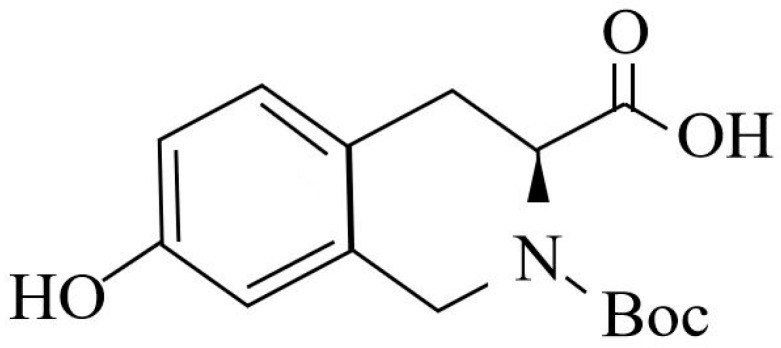
Structural formula of the isoquinoline compound in this study.

The isoquinoline compound is (s)-2-tert-butoxycarbonyl-7-hydroxy-1,2,3,4-tetrahydroisoquinoline-3-carboxylic acid (BHTCA), with the structural formula shown in Figure 1. BHTCA was synthesized by Shanghai Aladdin Biochemical Technology Co., Ltd., Shanghai, China.

The drug is composed of the following ingredients listed by volume: isoquinoline compound (*v*/*v* = 30%), Tween-80 surfactant (*v*/*v* = 5%), dimethylsulfone solvent (*v*/*v* = 10%), and sterile deionized water (*v*/*v* = 55%). The drug was prepared as an emulsion by first dissolving BHTCA and dimethylsulfone in a portion of sterile deionized water, followed by the addition of Tween-80 with vigorous stirring, and finally made up to the final volume with the remaining water.

### 2.2. Fish for Testing

Grass carp severely infected with *I. multifiliis* (collected from a limpid spring water fish farm in Kaihua County, Zhejiang province) were randomly selected for examination. Ten fish were sampled, and their gill tissues were observed under a microscope to count the number of *I. multifiliis* present. The infection rate of *I. multifiliis* among the experimental fish was 100%, with an average of approximately 80 parasites detected per fish.

### 2.3. In Vitro Drug Assay

Severely infected grass carp with *I. multifiliis* were transferred into a beaker. After allowing *I. multifiliis* to swim out, mature *I. multifiliis* were collected into a Petri dish using a pipette. Some of the collected parasites were utilized to cultivate *I. multifiliis* theronts, while another was used for tomont collection.

Different assay conditions were used for theronts and tomonts reflective of their distinct biological characteristics. Theronts are short-lived, free-swimming, and highly susceptible; thus, they were exposed to lower concentrations (0.05–0.3 mg/L) and observed over a shorter timeframe (up to 4 h). Tomonts, being reproductive cysts with protective membranes, are more resistant; therefore, they were tested with higher concentrations (0.1–0.5 mg/L) and observed over a longer period (24 h) to assess inhibition of division and hatching.

#### 2.3.1. In Vitro Efficacy Test of *I. multifiliis* Theront

The experiment was conducted using a 24-well cell culture plate, with approximately 2 mL of different concentrations of antiparasitic agent (0.05, 0.10, 0.15, 0.20, 0.25, and 0.3 mg/L) and approximately 100 *I. multifiliis* theronts added to each cell well. Observation under a microscope was performed at 15 min, 1 h, 2 h, 3 h, and 4 h after treatment, followed by the assessment of *I. multifiliis* mortality rates in each well after 4 h. The experimental setup included a well-ventilated natural water control group. Each experimental group was replicated three times.

Criteria for determining *I. multifiliis* mortality included immotility of cilia, cessation of cytoplasmic flow, rupture of the cell membrane, and fragmentation of the cell nucleus.

#### 2.3.2. Efficacy Testing of Drug Against *I. multifiliis* Tomont In Vitro

The experiment was conducted in 24-well cell culture plates, with approximately 2 mL of the drug at different concentrations (0.1, 0.2, 0.3, 0.4, and 0.5 mg/L) added to each well, along with 30 *I. multifiliis* tomonts. Microscopic observations were conducted 4 h after drug administration, and the mortality rate of *I. multifiliis* tomonts in each well was recorded 24 h later. The experiment included adequately aerated untreated water control groups, with each experimental group replicated three times.

Criteria for judging the death of *I. multifiliis* tomonts included tomonts remaining undivided or failing to hatch theronts.

### 2.4. Efficacy Testing of Drug Against I. multifiliis In Vivo

One hundred and twenty apparently healthy grass carp, showing no visible signs of parasitic infections, were placed in a 1 m^3^ water tank and temporarily raised for one week. Subsequently, 600,000 *I. multifiliis* theronts were introduced for infection. After 24 h of infection, the infected grass carp were grouped, with 10 fish in each group, and transferred to 192 L fish tanks. Based on preliminary range-finding experiments, three concentrations of the antiparasitic agent (0.2, 0.4, and 0.6 mg/L) were selected for the in vivo trial. Adequately aerated natural water with a pH of 7.0 to 7.5 and a temperature of 25 ± 1 °C was prepared in glass tanks measuring 80 cm × 60 cm × 40 cm. The designated concentrations of the drug were added to the water and thoroughly mixed. On the third and fifth days, new doses of the drug were added to each experimental group.

On the fifth day of the experiment, three grass carp from each concentration group were removed and placed in beakers. Tomonts were collected according to the method used in the in vitro acaricidal assay; mature trophonts (white spots) were gently scraped from the skin and fins of the sampled fish, and observations were made regarding the mortality and division of tomonts. Upon isolation from the host, the collected trophonts promptly develop into tomonts in aqueous conditions. Consequently, subsequent analyses were conducted on these newly formed tomonts to evaluate the drug’s efficacy.

On the tenth day of the experiment, all grass carp from each group were removed for sample collection. The entire gill apparatus was dissected and examined under a microscope to count the number of *I. multifiliis* present on the gills and on the fins of each fish. Parasite counts were conducted using a standardized protocol, with three randomly selected gill arches per fish examined and three fields of view per arch assessed. The average ciliates-killing rate was calculated as follows:

Ciliates-killing rate (%) = (Average number of *I. multifiliis* in the control group—Average number of *I. multifiliis* in the experimental group)/Average number of *I. multifiliis* in the control group × 100%.

Average fish mortality rate (%) = (Number of fish introduced − Number of fish surviving at the time of inspection)/Number of fish introduced × 100%.

### 2.5. Acute Toxicity Test of BHTCA Antiparasitic Agent on Grass Carp

The experiment was conducted in glass tanks with a volume of 80 L each. Ten healthy grass carp were placed in each tank, and the antiparasitic agent were added at concentrations of 5, 10, 15, 20, 25 and 30 mg/L. After 96 h, the number of dead grass carp was recorded, and the median lethal concentration (LC_50_) of BHTCA for grass carp was calculated. Three replicates were performed for each concentration group, with one control group included in the experiment.

### 2.6. Data Analysis

Data analysis was conducted using the statistical software SPSS 22.0. Statistical analysis was performed using one-way ANOVA followed by Tukey’s post-hoc test for multiple comparisons where appropriate. All parameters are expressed as mean ± standard deviation (M ± SD).

## 3. Results

### 3.1. In Vitro Efficacy of BHTCA Against I. multifiliis

**Figure 2:**
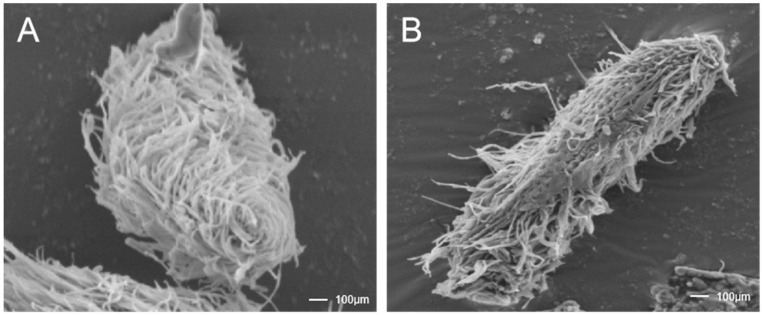
The antiparasitic agent BHTCA exhibits disruptive effects on the ciliary structure of the theront of *Ichthyophthirius multifiliis*. In the control group, cellular morphology appears normal, with cilia adhering to the surface at moderate density and exhibiting intact structure (**A**). In the BHTCA-treated group, cilia on the cell surface are shed, indicating damage to the ciliary structure (**B**).

The drug BHTCA demonstrates potent in vitro cidal activity against the theront stage of *I. multifiliis*, as depicted in Figure 2. The effectiveness of BHTCA increases with concentration, exhibiting enhanced lethality over time. The median effective concentrations (EC_50_) for mortality of *I. multifiliis* theronts at 15 min, 1 h, 2 h, 3 h, and 4 h are 0.26 (95% CI: 0.24–0.28), 0.22 (95% CI: 0.20–0.24), 0.18 (95% CI: 0.17–0.19), 0.15 (95% CI: 0.13–0.17), and 0.10 (95% CI: 0.08–0.12) mg/L, respectively. Concentrations of 0.25 mg/L and 0.3 mg/L achieved complete mortality within 4 h.

**Figure 3:**
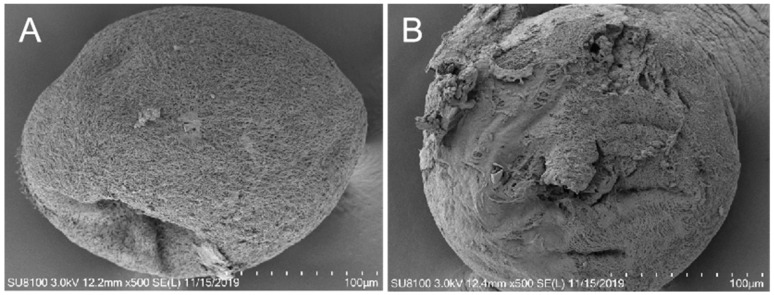
The drug BHTCA demonstrates potent in vitro cidal activity against the tomont of *Ichthyophthirius multifiliis*. In the control group, the capsule morphology appears normal, with a smooth surface (**A**). In the BHTCA-treated group, the capsule structure is disrupted, with membrane integrity compromised (**B**).

Microscopic examination revealed that normal theront cells maintain intact structures with orderly cilia, whereas cells treated with BHTCA showed compromised cellular integrity and cilia loss, as seen in Figure 2. Further investigation of BHTCA’s impact on the tomont stage is illustrated in Figure 3, with detailed results presented in Table 1. At a concentration of 0.5 mg/L, BHTCA prevented division and hatching of tomonts, achieving a 100% kill rate. Lower concentrations of 0.4, 0.3, and 0.2 mg/L resulted in mortality rates of 83.3%, 67.7%, and 43.3%, respectively. The least effective concentration, 0.1 mg/L, yielded a mortality rate of only 26.7% (Table 1).

The data indicate that BHTCA possesses significant in vitro lethal activity against both the theront and tomont stages of *I. multifiliis*, with efficacy positively correlated with the concentration of the drug. These findings suggest the potential of BHTCA in managing infection of *I. multifiliis* in aquatic environments.

Figure 3 illustrates that under normal conditions, tomont cells display a complete structure with a clear cell membrane. However, in the treatment group, the tomont cells are ruptured.

### 3.2. In Vivo Efficacy of BHTCA

The results of in vivo efficacy tests of BHTCA on grass carp parasitized by *I. multifiliis* are presented in Table 2. 

The table indicates that BHTCA substantially reduces the number of *I. multifiliis* on the body surface and gills of grass carp, along with a significant decrease in the hatching rate of theronts from fallen tomonts. At a concentration of 0.6 mg/L, the mortality rate of grass carp was 33.3%, with counts of *I. multifiliis* on the gills and fins being 112.8 ± 22.2. In contrast, the control group exhibited a 100% mortality rate, with *I. multifiliis* counts on the gills and fins of 516.2 ± 45.6. These results demonstrate that BHTCA provides significant protection to experimental fish infected with *I. multifiliis*.

The concentration (mg/L), tomonts collected on day 5, fish mortality rate (%), and the number of *I. multifiliis* on the fish body surface (gills and fins) are reported.

### 3.3. Acute Toxicity Test of BHTCA on Grass Carp

**Figure 4:**
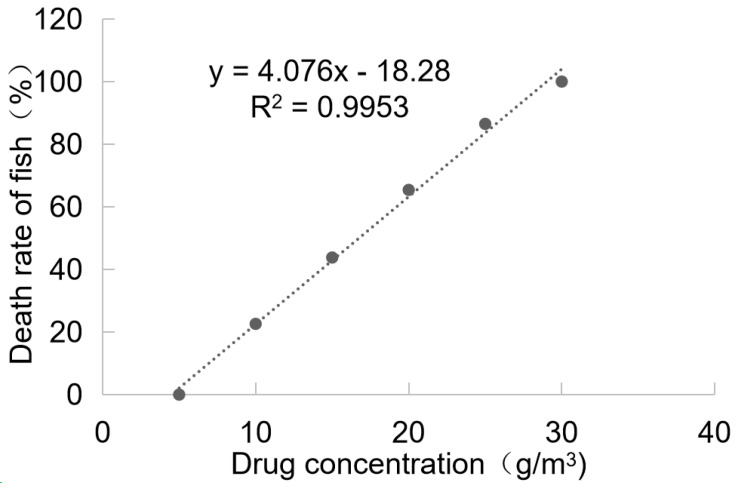
Toxicity results of BHTCA in grass carp. Relationship between changes in drug concentration and mortality rate of grass carp: y = 4.076x − 18.28, R^2^ = 0.9953.

The toxicity results of BHTCA in grass carp are depicted in Figure 4. At a concentration of 5.0 mg/L, no mortality was observed in grass carp after 96 h of treatment with BHTCA, and the fish exhibited normal activity without any adverse reactions. At a concentration of 30.0 mg/L, grass carp displayed symptoms of rapid breathing and jumping within 4 h of treatment, and all fish died within 96 h. The 96-hour LD50 (lethal dose for 50% of the population) of BHTCA for grass carp was determined to be 16.75 mg/L.

## 4. Discussion

Ichthyophthiriasis, commonly referred to as white spot disease, poses significant challenges to freshwater aquaculture and the aquarium industry [3,4,5,6,7,8,9,10,11]. In the life cycle of *I. multifiliis*, theronts are the infective stage for fish hosts [17,18]. Therefore, killing or inhibiting theronts will prevent the invasion of fish, which is essential to control white spot disease. Theronts, like most planktonic ciliates, are free-swimming in water. Although few studies have revealed the top-down impact on structure and compounds in the sanguinarine pathway [19,20], until now, few reports have been published about the effect of isoquinoline compounds on *I. multifiliis*.

Isoquinoline-type alkaloids show biological activities like those of morphinane-, protoberberine-, and benzophenanthridine-type alkaloids, and they are widely distributed in the plant kingdom, mainly in Papaveraceae, Berberidaceae, Ranunculaceae, and Menispermaceae. The production of some pharmaceutically interesting compounds from these plants by means of plant cell culture has been extensively studied [21].

Recent advances in pharmacological research have focused on developing safer, more effective treatments that reduce environmental impact. Studies exploring the use of natural compounds such as garlic, ginger, and various herbal extracts have shown promise in reducing *I. multifiliis* infection without the adverse effects associated with traditional chemicals [22]. Despite the availability of effective treatments, the emergence of drug-resistant strains of *I. multifiliis* poses a significant challenge. Continuous monitoring and the development of new therapies are essential. Future research should focus on the synergistic effects of combined treatments and the development of vaccines to provide long-term protection against this parasite.

The current research status on isoquinoline-derived synthetic drugs for antiparasitic applications remains somewhat exploratory but shows potential based on their pharmacological properties observed in related medical fields, particularly oncology and neurology [23,24]. Isoquinoline derivatives are known to interact with key enzymes and receptors involved in cellular processes. For instance, certain tetrahydroisoquinoline (THIQ) compounds inhibit phenylethanolamine N-methyltransferase (PNMT), suggesting a potential mechanism against parasitic infections via disruption of essential parasite enzymes [25]. Structural studies, including X-ray crystallography, have elucidated the binding mechanisms of these compounds to target proteins. Insights from complexes such as those with Bcl-2 proteins [26] can guide the design of isoquinoline-based antiparasitic agents targeting analogous pathways in parasites.

BHTCA demonstrates potent efficacy against *I. multifiliis* through targeted disruption of critical parasitic structures and life cycle transitions. In vitro observations reveal rapid disintegration of the theront ciliary apparatus (Figure 2B), directly impairing motility and host-invasion capabilities—key virulence determinants of this infective stage. This structural compromise correlates with concentration-dependent mortality (EC_50_ = 0.10–0.26 mg/L within 4 h), suggesting that BHTCA induces irreversible cytoskeletal damage that precedes cell death.

Based on the comparative analysis of the efficacy data, BHTCA demonstrates superior in vitro potency against the theront of *I. multifiliis* compared to both berberine and sanguinarine. The EC_50_ value of BHTCA was determined to be 0.10 mg/L after a 4-hour exposure, which is substantially lower than the EC_50_ of 7.86 mg/L reported for berberine [27] and the LC_50_ of 0.437 mg/L for sanguinarine [19]. This indicates that BHTCA is approximately 78 times more potent than berberine and about 4 times more potent than sanguinarine in killing the infectious theronts. Furthermore, BHTCA achieved 100% theront mortality at a concentration of 0.3 mg/L within 4 h, whereas berberine required 15 mg/L for a similar effect. This marked difference in effective concentrations suggests that the synthetic modification leading to BHTCA has resulted in a compound with greatly enhanced parasiticidal activity. The stage-selectivity of BHTCA is also noteworthy; while it was highly effective against theronts, its efficacy against the more resistant tomont stage (EC_50_ = 0.40 mg/L at 24 h) was still superior to berberine, which showed no direct lethal effect on protomonts even at 20 mg/L but could inhibit theront release at 5 mg/L.

BHTCA also exhibits a highly favorable therapeutic index, a critical consideration for aquaculture applications. The 96-hour LC_50_ of BHTCA for grass carp was 16.75 mg/L, resulting in a therapeutic index (LC_50_/EC_50_-theront) of greater than 167. This safety margin is considerably wider than that of berberine (LC_50_ = 528.44 mg/L, TI ≈ 67) and sanguinarine, for which a direct LC_50_ in grass carp was not provided by Yao et al., but is implied to be safe at effective concentrations below 0.9 mg/L. The high therapeutic index of BHTCA, coupled with its low effective concentration, suggests a potentially greater margin of safety for treated fish and the surrounding aquatic environment. The proposed mechanism of action for BHTCA, involving the disruption of ciliary structures and cyst integrity, aligns with the physical degradation observed in sanguinarine-treated parasites (e.g., destruction of the outer cell membrane and macronucleus), but appears to occur at a much lower concentration. This combination of high potency, stage-specific efficacy, and wide safety margin positions BHTCA as an exceptionally promising candidate for the sustainable control of ichthyophthiriasis, warranting further investigation into its precise molecular targets and large-scale field trials.

Investigations into the effects of isoquinoline derivatives on gene expression also provide a foundation for their antiparasitic potential. Studies have shown that these compounds can induce apoptosis and inhibit cell proliferation in vitro [28,29], which are desirable effects for compounds targeting parasites that rely on rapid replication and protein synthesis to maintain infection.

While direct applications in antiparasitic therapy are not yet extensively documented, ongoing research in related areas lays the groundwork for future studies. Continued exploration of isoquinoline derivatives’ biochemical interactions, molecular targets, and cellular effects will be essential to establish their efficacy and safety as antiparasitic agents. Future research should focus on specific parasitic models and the development of compounds that are both effective against parasites and safe for human use.

In conclusion, pharmacological control remains a vital component in managing *I. multifiliis* infections in freshwater fish. This study represents the initial discovery of BHTCA exhibiting potent antiparasitic properties against *I. multifiliis*. The stage-selective susceptibility (theronts > tomonts) indicates that BHTCA likely targets cilia-associated proteins, a hypothesis that merits validation through proteomic analysis. Within the context of freshwater fish aquaculture, its toxicity concentration in grass carp exceeds 80 times the minimum effective drug concentration, indicating a considerable margin of safety at higher dosages. The preparation of this novel antiparasitic agent involves a straightforward process, resulting in low production costs. It demonstrates both high efficacy and safety in application, while being environmentally friendly. Moreover, this compound exhibits significant efficacy in the eradication of *I. multifiliis*. This discovery presents a promising avenue for the control of parasitic organisms in freshwater aquaculture.

## Figures and Tables

**Table 1:** Effect of BHTCA substance on the mortality of *I. multifiliis* tomonts and the inhibition of hatching (6 h).

Drug Concentration (mg/L)	Death Rate of Tomonts (%)	Total Number of Hatched Tomonts
0.1	26.7 ± 6.7 ^b^	589.5 ± 25.6 ^a^
0.2	43.3 ± 6.7 ^c^	418.5 ± 36.5 ^b^
0.3	67.7 ± 3.3 ^d^	269.2 ± 29.8 ^c^
0.4	83.3 ± 6.7 ^e^	144.3 ± 33.7 ^d^
0.5	100 ± 0.0 ^f^	0.0 ± 0.0 ^e^
control	0.0 ± 0.0 ^a^	603.5 ± 32.5 ^a^

Within a column, different superscript letters denote statistically significant differences analyzed by Tukey’s test (*p* < 0.05).

**Table 2:** In vivo efficacy of BHTCA substance against *I. multifiliis*.

Drug Concentration (mg/L)	Tomonts Collected on 5th Day		Death Rate of Fish (%)	Total No. of *Ich* (Gill and Fins)
	Death Rate (%)/Total Number of Hatches			
0 (control)	0.0 ± 0.0 ^a^	595.5 ± 53.3 ^a^	100.0	516.2 ± 45.6 ^a^
0.2	36.7 ± 6.7 ^b^	585.2 ± 48.8 ^a^	70.0	518.4 ± 64.7 ^a^
0.4	63.3 ± 3.3 ^c^	414.3 ± 36.5 ^b^	40.0	245.3 ± 55.6 ^b^
0.6	76.7 ± 6.7 ^c^	366.7 ± 49.2 ^b^	33.3	112.8 ± 22.2 ^c^

Within a column, different superscript letters denote statistically significant differences analyzed by Tukey’s test (*p* < 0.05).

## Data Availability

Data are contained within this article.
